# Supplementary material for: High-fidelity remote entanglement of trapped atoms mediated by time-bin photons
Source: Nat Commun. 2025 Mar 14;16:2533. doi: 10.1038/s41467-025-57557-4 (PMC11909202; doi:10.1038/s41467-025-57557-4)
Supplement: Supplementary file 1 — Supplementary Information [file 41467_2025_57557_MOESM1_ESM.pdf]

Supplementary Information

Beam geometry and Lamb-Dicke Parameters for optimal cooling

The geometry of the input beams and emitted photons for the traps is shown in the schematic, Fig. 1. The excitation and cooling beams at 493 nm are delivered colinearly at an angle of $\beta_q = 45°$ degrees to the axial (z) axis of both traps. The emission direction is perpendicular to the z-axis and at an angle α_q to each trap's principal x-axis.

We are interested in the angles θ_{qi} between the excitation/cooling wavevectors and the principal axes i as well as the angles ψ_{qi} between the emission wavevector and the principal axes:

$$\cos\theta_{qx} = -\sin\beta_q \sin\alpha_q \qquad \cos\psi_{qx} = \cos\alpha_q \tag{1}$$

$$\cos\theta_{qy} = \sin\beta_q \cos\alpha_q \qquad \cos\psi_{qy} = \sin\alpha_q \tag{2}$$

$$\cos\theta_{qz} = -\cos\beta_q \qquad \cos\psi_{qz} = 0. \tag{3}$$

Hence, the Lamb-Dicke recoil parameters are given by

$$\eta_{qi} = \sqrt{\frac{\hbar k^2}{2m\omega_{qi}}}|\cos\psi_{qi} - \cos\theta_{qi}| \tag{4}$$

$$\zeta_{qi} = \sqrt{\frac{\hbar k^2}{2m\omega_{qi}}}|\cos\psi_{qi}| \tag{5}$$

and the expected Doppler cooling limit is(1)

$$\bar{n}^{\mathrm{D}}_{qi} = \frac{\gamma}{4\omega_{qi}}\left[\frac{\Delta}{\gamma} + \frac{\gamma(1+s)}{4\Delta}\right]\left(1 + \frac{1}{3\cos^2\theta_{qi}}\right). \tag{6}$$

Here $\Delta \sim \gamma/2$ is the red detuning of the Doppler cooling beam from resonance and $s = I/I_{\mathrm{sat}} \sim 2$ is the saturation parameter with I the laser intensity and I_{sat} the saturation intensity. We note that one particular principal axis in Bob (B) is nearly orthogonal to the cooling beam ($\theta_{By} \approx 87°$). This single direction has a poor Doppler cooling limit and hence is the dominant source of error as seen in the section on atomic recoil in the main paper. We estimate an uncertainty in all geometrical angles to be $< 3°$.

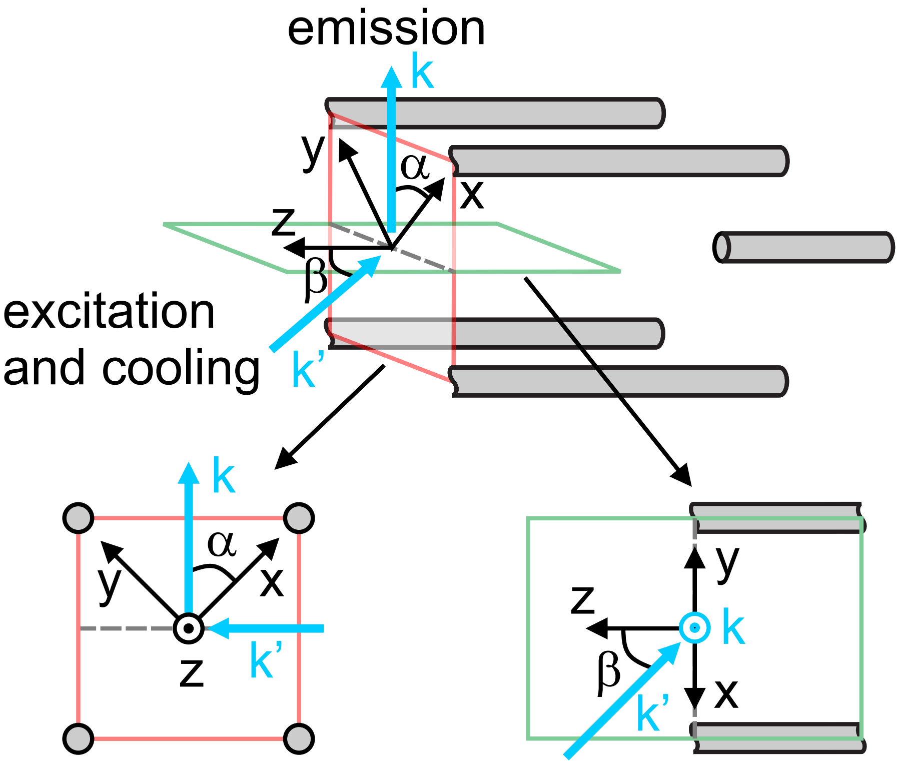

Supplementary Figure 1: The orientation of trap principal axes x, y and z with respect to pulsed excitation, Doppler cooling and single photon emission directions (see Table 1). The excitation and Doppler cooling wavevector k' is perpendicular to the emission wavevector k.

Supplementary Table 1: Summary of beam angles, Lamb-Dicke recoil parameters, and Doppler cooling limits $\bar{n}_{qi}^{\mathrm{D}}$ for each motional normal mode i of ion q.

q	i	$\frac{\omega_{qi}}{2\pi}$ (kHz)	α_q	β_q	θ_{qi}	ψ_{qi}	η_{qi}	ζ_{qi}	$\bar{n}_{qi}^{\mathrm{D}}$
A	z	991.5	-	45°	135°	90°	0.055	0	13
A	x	1157.5	45°	45°	120°	45°	0.086	0.051	15
A	y	1488.0	45°	45°	60°	45°	0.013	0.045	12
B	z	330.3	-	45°	135°	90°	0.095	0	38
B	x	826.7	85.5°	45°	134.8°	85.5°	0.066	0.0067	15
B	y	992.0	85.5°	45°	86.8°	4.5°	0.073	0.077	826

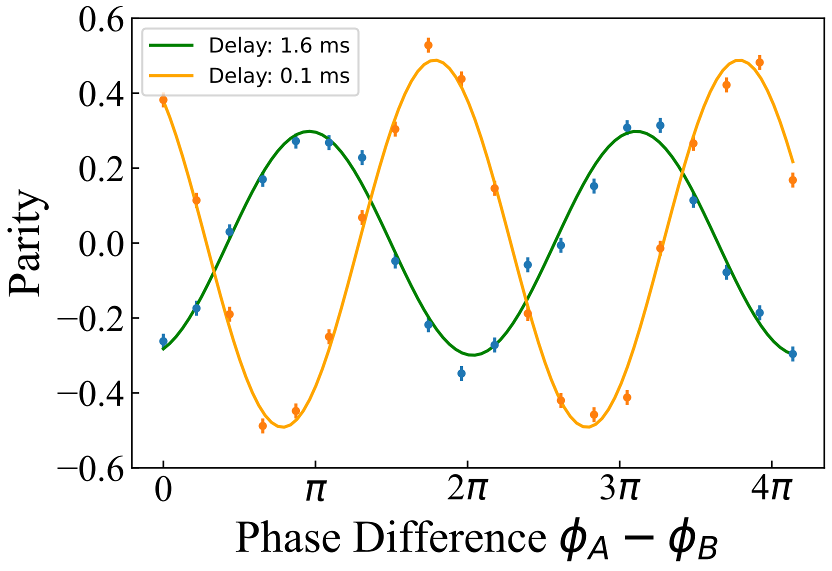

(a)

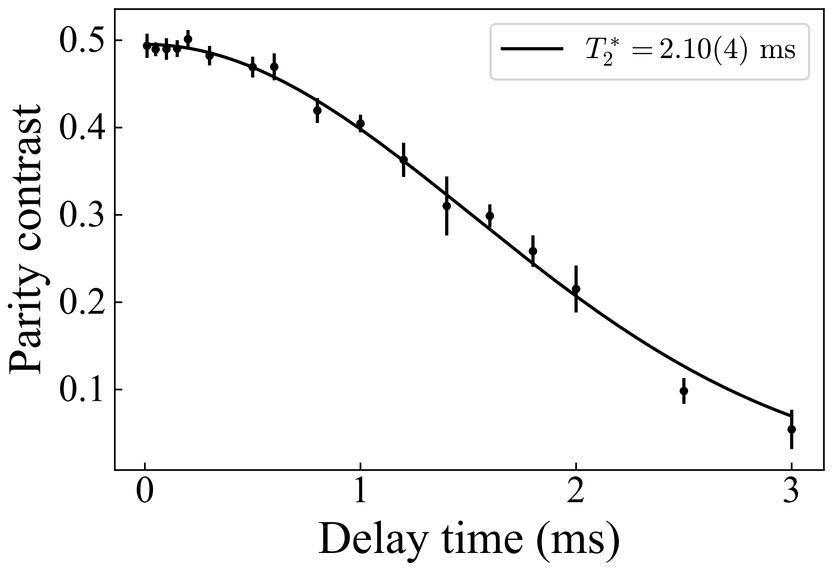

(b)

Supplementary Figure 2: Measurement of differential qubit coherence time between the qubits in Alice and Bob. (a) Measured oscillations of the two qubit parity with different relative phase for delay times of 0.5 ms and 1.6 ms. (b) Parity contrast as we vary the delay time between two $\pi/2$ pulses. Because the two ions are not entangled, a maximum parity contrast of 0.5 is expected. The fit to a Gaussian envelope gives a $1/e$ coherence time of $T_2^* = 2.10(4)$ ms.

Supplementary References

[1] D. J. Wineland and Wayne M. Itano. Laser cooling of atoms. *Phys. Rev. A*, 20:1521–1540, Oct 1979.
